# *Dictyostelium discoideum*: A Model System for Neurological Disorders

**DOI:** 10.3390/cells11030463

**Published:** 2022-01-28

**Authors:** Claire Louise Storey, Robin Simon Brooke Williams, Paul Robert Fisher, Sarah Jane Annesley

**Affiliations:** 1 Department of Microbiology, Anatomy, Physiology and Pharmacology, La Trobe University, Bundoora 3086, Australia; C.Storey@latrobe.edu.au (C.L.S.); P.Fisher@latrobe.edu.au (P.R.F.); 2 Centre for Biomedical Sciences, School of Biological Sciences, Royal Holloway University of London, Egham TW20 0EX, UK; robin.williams@rhul.ac.uk

**Keywords:** *Dictyostelium discoideum*, Parkinson’s Disease, Alzheimer’s Disease, Huntington’s Disease, lissencephaly, neuronal ceroid lipofuscinoses, neurological disorders, model organisms

## Abstract

Background: The incidence of neurological disorders is increasing due to population growth and extended life expectancy. Despite advances in the understanding of these disorders, curative strategies for treatment have not yet eventuated. In part, this is due to the complexities of the disorders and a lack of identification of their specific underlying pathologies. *Dictyostelium discoideum* has provided a useful, simple model to aid in unraveling the complex pathological characteristics of neurological disorders including Alzheimer’s disease, Parkinson’s disease, Huntington’s disease, neuronal ceroid lipofuscinoses and lissencephaly. In addition, *D. discoideum* has proven to be an innovative model for pharmaceutical research in the neurological field. Scope of review: This review describes the contributions of *D. discoideum* in the field of neurological research. The continued exploration of proteins implicated in neurological disorders in *D. discoideum* may elucidate their pathological roles and fast-track curative therapeutics.

## 1. Introduction

Neurological disorders are the predominant cause of disability and the second leading cause of death worldwide [1]. In the past 30 years mortality attributed to neurological disorders has risen by ~39% largely due to increased population and aging [1]. Mainly due to the inaccessibility of the human brain, insights into complex neurological disorders rely heavily on the development of model systems [2]. These model systems include human primary and immortalised cell lines, animal models (encompassing mammals—mouse and rat; nonmammalian vertebrates—zebrafish; invertebrates—nematodes and vinegar flies) as well as simpler eukaryotic models (e.g., yeast and *Dictyostelium discoideum*) [3,4,5,6,7,8]. All of them have fully sequenced genomes, but the simple eukaryotes are unsurpassed in their short life cycles, low cost, ease of clonal growth and genetic manipulation. These benefits make them well suited for screening, testing and developing therapeutic agents. Despite having no central nervous system, their experimental tractability provides advantages for studying the function of disease-associated genes and underlying cytopathological pathways. They are amongst a small cohort of organisms recognized by the NIH for their usefulness as biomedical models [9].

*D. discoideum* has all the usual genetic and experimental tractability of an established, simple eukaryotic model, but in addition, it has a unique life cycle which, depending upon nutrient availability can be either solitary or social and provides diverse phenotypic “readouts” of underlying cytopathological pathways. When nutrients are plentiful the amoebae remain solitary, however, upon nutrient deprivation they differentiate and then release pulses of the chemoattractant cAMP which induces the aggregation of ~100,000 cells into mounds [10]. The cells then further differentiate, resulting in two distinct populations of cells—prestalk and prespore cells which participate in multicellular morphogenesis and further differentiation to ultimately form a fruiting body. This exceptional lifecycle distinguishes *D. discoideum* from other microbial models and has provided an exciting, simple, cost-effective, non-sentient model organism for studying complex biochemical pathways underpinning neurological disorders [11].

The genome of *D. discoideum* has been entirely sequenced [12] and many orthologs of human genes associated with neurological disorders have been identified. The genetic tractability of the organism allows genes related to such disorders to be easily manipulated and phenotypically analysed allowing insight into their conserved cellular functions. In addition to altering endogenous genes, several human genes have been expressed in *D. discoideum* and studied in the absence of endogenous copies, for example, human α-synuclein and Tau protein have been expressed singly and in combination in *D. discoideum* to elucidate the mechanisms of cellular toxicity in synucleinopathies and tauopathies [13,14]. Furthermore, many cellular processes are highly conserved in *D. discoideum* allowing for the investigation of underlying cytopathological mechanisms. For example, *D. discoideum* does not contain a homolog of human amyloid precursor protein (APP) but it has been expressed in *D. discodeum* and shown to be processed as in humans to produce amyloid beta (Aβ) peptides and other APP metabolites [15].

Despite lacking a central nervous system, *D. discoideum’s* highly conserved cellular processes have allowed it to provide much insight into key cellular abnormalities associated with neurological disorders such as mitochondrial dysfunction and aberrant lysosomal activity [16,17]. This review will highlight how *D. discoideum* has been used to study some of the most common neurodegenerative diseases including Parkinson’s disease (PD), Alzheimer’s disease (AD), Huntington’s disease (HD), neuronal ceroid lipofuscinoses, and lissencephaly [18]. Furthermore, *D. discoideum* has been used for elucidating the underlying mechanisms of action of therapeutic pharmacological agents and the screening of novel compounds prior to validation in higher eukaryotes and this review will highlight some of the main studies in this area [19,20,21].

## 2. Alzheimer’s Disease (AD)

AD is the most common form of dementia, accounting for around 75% of cases [22], and the incidence is expected to triple by 2060 [23]. The central pathological characteristics of AD are the accumulation of neurofibrillary tangles comprised of hyperphosphorylated Tau proteins and of plaques comprised of amyloid-β proteins. These two key features have been hotly debated as the origin of the disease [24]. Support for AD pathology relating to plaque formation was initially advanced by the identification of mutations in patient populations with an early onset (familial) form of AD [25], arising from a range of loss-of-function mutations in components of the γ-secretase complex, especially in two presenilin proteins (Psen1 and Psen2), and in the amyloid precursor protein (APP) that is cleaved by the γ-secretase complex to form amyloid-β proteins. Research into AD pathology and treatment has therefore focused on the processes leading to the formation of tangles and plaques [26], but this has provided limited advances in treatments, suggesting that improved understanding of the role of the γ-secretase complex and presenilin proteins beyond tangle and plaque formation may provide alternative approaches for treatment.

*D. discoideum* provides a tractable model system, with a well-conserved γ-secretase complex including two presenilin proteins, where single and multiple genes encoding these proteins can be easily ablated and resulting isogenic mutants analysed. Furthermore, the model does not form tangles and plaques, allowing the effect of loss of function γ-secretase complex components to be investigated independent of tangle and plaque formation [15,27,28,29]. The key complex components (Aph1, Ncst1, Pen2 and two presenilin proteins) in *D. discoideum* are of similar size, domain structure and catalytic amino acids to those found in mammals, suggesting conserved function. Analysis of the role of these individual components initially revealed that loss of both of the *D. discoideum* presenilin proteins (PsenA and PsenB), interrupts the process of multicellular development, to provide a clear and easily assessed phenotype for monitoring presenilin function [27]. The relevance of this model system and assay to the human condition is demonstrated by the rescue of this developmental block through the expression of the human Psen1 protein in the double mutant, confirming the functional homology of human and *D. discoideum* presenilin proteins. Interestingly, the presence of the key catalytic aspartic acid residues on either the *D. discoideum* PsenB (D348A and D394A) or the human Psen1 (D257A and D358A) is not required for developmental rescue [27,28], indicating a non-proteolytic function for both proteins in development. However, both human Psen1 and Psen2 retain auto-proteolytic activity in *D. discoideum*, through the cleavage of the human presenilin proteins to yield short C-terminal fragments [27], although the endogenous substrates remain to be identified. Development is not compromised by the loss of the other γ-secretase components, highlighting key roles for presenilin proteins rather than the γ-secretase in development in this model. Fluorescent tagging of the *D. discoideum* γ-secretase complex components (PsenB, Ncst, and Aph1) localised the complex to the endoplasmic reticulum, consistent with what is found in mammalian models [30]. Thus, presenilin proteins control development in *D. discoideum*, and this cellular function is conserved in the human homologues, evidencing *D. discoideum* as a suitable model to investigate human γ-secretase complex and presenilin protein function.

Further research into presenilin and the γ-secretase activity in *D. discoideum* identified a key role for the γ-secretase complex in regulating autophagy, in a proteolytic independent mechanism. Since the γ-secretase in mammalian models regulates endocytosis [31,32,33], and in *D. discoideum* regulates phagocytosis [15], a role for this complex was further examined in *D. discoideum*. Here, the dominant form of liquid uptake is via macropinocytosis, and this was shown to be reduced in a mutant lacking a component of the γ-secretase (Aph1) or both presenilin proteins [28], suggesting a key role for this complex is conserved in *D. discoideum*. Furthermore, reduced macropinocytosis following the loss of both presenilin proteins was restored through the expression of the human Psen1 protein, confirming evolutionary conservation of these proteins in this role. Surprisingly, the two key catalytic aspartic acids necessary for proteolytic activity are also not necessary for this endocytosis activity. Further analysis identified the role of the γ-secretase complex (including the presenilin proteins) in phagosomal proteolysis and autophagic flux, in regulating the time to acidification of the autophagosome, and thus reduced activity of the protein recycling complex in autophagy.

Thus, in *D. discoideum*, the function of presenilin proteins and the γ-secretase complex in regulating development and autophagy are independent of presenilin proteolytic activity. The activity of presenilin proteins in this proteolytic independent activity is also conserved in the human presenilin 1 protein. These data, therefore, suggest that presenilin proteins, and to a lesser extent the γ-secretase complex, function to regulate autophagy, phagosomal proteolysis and autophagic flux, in a manner that is independent of proteolytic cleavage activity. This role may be further investigated in relation to pathogenic changes in AD.

## 3. Parkinson’s Disease (PD)

Parkinson’s disease is the leading cause of motor dysfunction and the second most prevalent neurodegenerative disorder after Alzheimer’s disease [34]. PD is characterised by motor symptoms including tremor, bradykinesia, rigidity, and gait abnormalities and non-motor clinical symptoms including sleep, behavioural disturbances and cognitive decline [34,35]. A defining hallmark of the disease is the loss of dopaminergic neurons from the *substantia nigra* region of the midbrain and the inclusion of Lewy bodies in surviving neurons [36].

Most cases of PD are sporadic or idiopathic with no known underlying genetic cause [37]. There are, however, rarer cases (about 10% of all PD) termed familial or genetic PD in which mutations to a single gene have been identified as the cause and many of these genes appear to regulate common cellular pathways such as mitochondrial signalling and lysosomal degradation. By genetically manipulating *D. discoideum,* various PD models have been created which alter the levels of one or more of the gene products known to be associated with PD. These models include manipulation of homologs of PD-associated genes (listed in Table 1) and also the expression of human PD-associated genes which do not have homologs in *D. discoideum* (Table 1). These models have been used to help unravel the complex pathophysiological mechanisms underlying PD and to better understand why certain mutations or repetitions give rise to the disease. Each of these PD-associated gene models will be discussed in further detail below.

### 3.1. Leucine Rich Repeat Kinase 2 (LRRK2)

Mutations in *LRRK2* are the most common genetic cause of autosomal dominant, late-onset PD and genome-wide associated studies (GWAS) have subsequently linked the gene to sporadic cases of PD [47]. LRRK2 is a member of the Roco protein family, first discovered in *D. discoideum* in 2002 by Goldberg et al. [48]. The exact role LRRK2 plays in the pathogenesis of PD remains elusive, as does its normal role in healthy cells. There are 11 Roco family members in *D. discoideum* [38], more than is known in any other organism. These Roco proteins have diverse functions including roles in chemotaxis, development and growth. Roco1, also known as cyclic GMP-binding protein C (GbpC), plays a central role in chemotaxis. Upon starvation, and the subsequent release of the chemoattractant cAMP, Roco1 translocates to the cell membrane and interacts with myosin II [46]. Myosin II is regulated by phosphorylation and is essential for chemotaxis and cell polarity. Roco1 null mutants have abnormally phosphorylated myosin II and this cements a role for Roco1 in chemotaxis [15].

Like Roco1, Roco2/Pats1, also functions in chemotaxis wherein null mutants aggregate more slowly into larger mounds [39]. In addition, Pats1 has a role in cell division as null mutants display defects in cytokinesis [40]. Roco3/QkgA plays a role in cell proliferation with null mutants growing quicker in shaking culture and strains overexpressing QkgA growing slower [41]. Null mutants of Roco4 and Roco11 display aberrant fruiting body morphology revealing that both proteins are essential to normal multicellular development [43].

It is unclear which of the *D. discoideum* Roco kinases is the functional homolog of human LRKK2, but Roco4 has the highest combined sequence and structural similarity and in addition to the core LRR, Roc and kinase domains it also contains a WD40 domain like LRRK2 [42]. Specific human LRKK2 pathogenic variants are rare and often confined to a few families, however, the most studied mutation, a substitution at residue 2019 (G2019S) in the kinase domain, accounts for ~4% of familial and ~1% of sporadic cases worldwide [49]. This G residue is conserved in *D. discoideum* Roco4 at residue 1179 and the equivalent mutant form has been created (G1179S) and expressed in the Roco 4 null background [43]. As in human LRRK2, mutation of this residue in Roco 4 results in increased kinase activity in vitro [42]. Roco4 plays a key role in the *D. discoideum* developmental cycle. It is expressed throughout multicellular development with a peak in expression at the multicellular slug stage [38,42]. Its ablation results in normal aggregation but delayed tip formation and subsequent multicellular development with culmination occurring after 70 h compared to 12 h in the wild type (WT). The Roco4 null mutant also displayed a reduction in the number of fruiting bodies that formed and had a severe defect in the fruiting body morphology with very few stalk cells [43]. The reduction in stalk cells was determined to be due to a lack of cellulose rather than a defect in the stalk differentiation pathway [38]. The lack of cellulose produces unordered stalk cells which are unable to form a rigid structure and cannot support the sorus resulting in sori appearing to form on the agar surface [38]. The developmental defect was rescued by ectopic overexpression of the wild type Roco4 protein, or its kinase domain only, or a chimeric Roco4/Lrrk2 protein in which the Roco4 kinase domain was replaced with the human LRRK2 kinase domain. However, the mutant Roco4^G1179S^ protein only partially rescued the developmental defect in the null background [43]. Although the Roco4^G1179S^ strains had no defect in the number of fruiting bodies, their development was still significantly delayed, and their morphology was aberrant with smaller stalks. At the multicellular slug stage Roco4 null slugs did not migrate and Roco4^G1179S^ strains had impaired phototactic orientation towards the light source. In both strains, these defects were rescued by the expression of the Roco4 wild type protein [43].

Given that phototactic defects can often manifest due to impaired mitochondrial function [50] and that human LRKK2 is implicated in mitochondrial function, this was assayed directly using two measures of mitochondrial function—reactive O_2_ species (ROS) levels (determined fluorometrically) and mitochondrial oxidative phosphorylation (using live cell respirometry in a Seahorse Extracellular Flux analyser) [51]. WT and Roco4^−^ strains displayed similar amounts of ROS, however after the addition of the protonophore CCCP, the Roco4^−^ mutant had elevated ROS compared to WT, indicating the Roco4^−^ mutant had a reduced ability to remove ROS following mitochondrial uncoupling [43]. Alternatively, the null mutant may have a greater capacity to generate ROS after CCCP uncoupling because of its elevated complex I activity under these conditions.

Respirometry experiments revealed basal oxygen consumption rates (OCR), OCR contributing to ATP synthesis and OCR by Complex I, were all elevated in the Roco4^−^ mutant. In contrast, basal OCR was significantly reduced in Roco4^G1179S^ strains. Interestingly, in the Roco4 wild type rescue and Roco4^G1179S^ strains maximum, OCR and spare capacity OCR were significantly reduced [43]. Electron microscopy revealed no defects in mitochondrial morphology or structure and no reduction in the number of mitochondria in the *D. discoideum* Roco4^−^ mutant. [43]. This suggests that the increased kinase activity present in the point mutant results in the impaired mitochondrial function which is in agreement with a gain of function property of the PD-associated mutation.

### 3.2. HTRA2 or Omi Protease

HTRA2, or Omi protease, is a nuclear-encoded protein that, in humans, primarily resides in the intermembrane space of the mitochondria [52]. It is a serine protease chaperone that is released to the cytosol prior to apoptosis and cleaves Inhibitor of Apoptosis Proteins (IAPs), initiating the apoptotic cascade [53]. Mutations in the gene are associated with late-onset autosomal dominant PD and it has been suggested that this is due to loss of HTRA2′s neuroprotective role [54]. Missense mutations in *HTRA2* lead to reduced protease activity, resulting in an accumulation of unfolded proteins within the mitochondria, eventually leading to mitochondrial dysfunction [55]. HTRA2 is also regulated by PINK1, a putative mitochondrial protein kinase, wherein PD-associated mutations in *PINK1* lead to reduced phosphorylation of HTRA2. Interestingly, when HTRA2 is overexpressed, this too can result in mitochondrial defects and unregulated apoptosis [53].

Mitochondrial dysfunction has been implicated as the cause of neuronal death in PD patients with reduced HTRA2 activity but the low penetrance of *HTRA2* and the fact that previously reported pathogenic variants have been discovered in seemingly healthy asymptomatic individuals does not support this [44]. These discrepancies may be explained by the complex phenotype–genotype relationship that is often described in mitochondrial biology.

To understand the normal and pathogenic roles of HTRA2 *D. discoideum* has been employed. A single homologue of *HTRA2* was identified in *D. discoideum* (*htrA*) and as in humans, the protein was shown to localise to the mitochondria [44]. Antisense inhibition of *htrA* revealed that reduction of HTRA2 as suggested by the reduced mRNA levels of HTRA2, partially phenocopied *D. discoideum* mitochondrial disease models with aberrant multicellular development and reduced growth rates with no corresponding defect in endocytosis [44]. As its protease activity has been implicated in its neuroprotective role the authors created strains that overexpressed a protease-dead HTRA2 protein with an amino acid substitution at residue 300 in the kinase domain (S300A). HTRA2^S300A^ expressing strains displayed phenotypes resembling the HTRA2 antisense-inhibited strains, including deranged fruiting body morphology and reduced growth rates in the absence of defects in endocytosis. This suggested that HTRA2′s role in these phenotypes could be ascribed to loss of the protease activity. The authors measured mitochondrial respiration directly via Seahorse respirometry and no defect in either the antisense-inhibited or S300A-expressing strains was detected. Overexpression of HTRA2 proved lethal to *D. discoideum* suggesting hyperactivity of the protein is also cytotoxic. The study revealed that HTRA2 in *D. discoideum* has a similar role to that in humans where tight regulation of HTRA2 is needed to avoid cytopathological dysfunction. Due to these similarities, *D. discoideum* may be useful in further elucidating HTRA2′s exact role in PD including its role in mitochondrial function. This study has highlighted the importance of HTRA2′s protease activity and how the loss of this activity in the mitochondria can result in cytopathological outcomes, even in the absence of measurably impaired mitochondrial respiratory function [44]. It is possible that other mitochondrial functions are more sensitively impaired by loss of HTRA2 function than is respiratory oxidative phosphorylation.

### 3.3. DJ-1

Unlike *HTRA2*, mutations in *DJ-1* (*Park7*) have extremely high penetrance despite being very rare [47]. Mutations in *DJ-1* account for 1% of early-onset PD and are autosomal recessive [56]. DJ-1 has also been implicated in idiopathic late-onset PD, whereby post-mortem analysis of PD brains has shown that the protein is more abundant and oxidatively damaged [45]. Interestingly, mutations in *DJ-1* appear to cause an increase in the clinical presentation of non-motor PD symptoms such as anxiety, psychosis, and cognitive decline [57]. DJ-1 appears to be particularly important to high energy-demanding tissues, such as the brain, where reactive oxygen species are generated more readily [58]. DJ-1 has multiple reported cellular roles, as a molecular chaperone, antioxidant, and protease, as well as ROS scavenger and transcriptional regulator. A conserved cysteine residue at position 106 is vital to DJ-1-mediated protection against oxidative stress [58]. DJ-1 has been shown to share common biological roles with PINK1, another protein that protects cells against mitochondrial dysfunction. In *Drosophila*, DJ-1 can rescue PINK1 knockout-associated phenotypes despite no substantial evidence for a direct interaction, indicating they share a common biological pathway [59].

DJ-1 also interacts with other PD-associated proteins such as α-synuclein, reducing its toxicity by sequestering α-synuclein monomers preventing its aggregation, however, DJ-1 can also act on α-synuclein fibrils and remodel these into toxic oligomers [60]. DJ-1 can directly regulate ROS production by increasing the expression of mitochondrial uncoupling proteins (UCP4 and 5) leading to reduced mitochondrial membrane potential and lower ROS production [58]. This has led to the conclusion that DJ-1 has potential roles in both neurodegeneration and neuroprotection [58,60]. DJ-1 acts as a sensor for oxidative stress and a molecular chaperone, but the mechanism by which DJ-1 achieves this is unclear [58,60].

DJ-1 has been reported to localise in the nucleus, mitochondria and cytoplasm [61]. Despite many studies, the exact function and localisation of DJ-1 remain uncertain [45]. *D. discoideum* has a single homologue of mammalian DJ-1, encoded by *deeJ*, and has been found to localise predominately to the cytoplasm. DJ-1 plays a vital role in multicellular development in *D. discoideum*. Antisense-inhibited strains exhibit aberrant fruiting body morphology with shorter, thicker stalks. This suggests that more cells are undergoing autophagic cell death and that DJ-1 may play a role in the regulation of autophagy. DJ-1 also has a role in growth and endocytosis with knockdown strains displaying reductions in growth in liquid media and on bacterial lawns. In both cases, the reduced growth was attributed to a reduced endocytic rate, reduced pinocytosis for liquid growth and reduced phagocytosis for growth on bacterial lawns. The converse was observed in DJ-1-overexpressing strains with increases in growth and endocytic rates [45].

Interestingly, studies performed in primary cortical neurons of *DJ-1* KO mice have revealed that synaptic vesicle endocytosis is severely impaired [62]. Similar results were also achieved using *DJ-1* KO mouse primary astrocytes, further validating DJ-1′s positive regulation of endocytosis [63]. *D. discoideum* is a well-established model for the study of endocytic pathways, with many endocytic proteins having already been characterised [64]. This provides an opportunity to properly characterise DJ-1′s role in regulating endocytosis and further elucidate its role in humans.

Despite the cytoplasmic localisation of DJ-1 in *D. discoideum* and its positive role in endocytosis, when overexpressed, DJ-1 has been shown to inhibit certain parameters of mitochondrial respiration [45]. Seahorse Extracellular Flux experiments revealed that, in DJ-1-overexpressing strains, basal and uncoupled respiration were reduced, as were ATP synthesis and Complex I activity. Conversely, the knockdown of DJ-1 slightly enhanced these parameters. Regression analysis revealed that both ATP synthase and Complex I are functionally normal in strains expressing altered levels of DJ-1, however, the total amount of respiratory activity was altered. These results are inconsistent with the proposed role of DJ-1 in protecting mitochondria, but are consistent with the elevated respiration rates also observed in other PD models including lymphoblasts and fibroblasts from idiopathic PD patients and in a neuroblastoma model exposed to α-synuclein fibrils [65,66,67]. Together this suggests that mitochondria may play a more complex role in the cytopathology of PD and warrants further investigation.

To investigate if DJ-1 played a role in protection against oxidative stress in *D. discoideum,* cells with altered expression of DJ-1 were exposed to H_2_O_2_ [46]. Like DJ-1 knockdown, exposure of wild type cells to H_2_O_2_ also caused impaired morphogenesis, growth and endocytosis, but the combination of DJ-1 knockdown and oxidative stress caused more severe defects than either on their own. Neither oxidative stress, nor DJ-1 knockdown caused a defect in slug phototaxis, but in combination, a dramatic defect in slug phototactic accuracy was produced. Taken together these results show that, as in other model organisms and in humans, DJ-1 provides cytoprotection against the effects of oxidative stress.

To understand how DJ-1 might exert these cytoprotective effects, it is important to understand the cytopathological mechanisms that are elicited by oxidative stress. Chen et al. [46] explored the possibility that AMP-activated protein kinase (AMPK) may act downstream of oxidative stress in the DJ-1 knockdown strains [46,68]. AMPK is known to play a role in the impairment of phototaxis and other phenotypes caused by mitochondrial dysfunction and may play a similar role after oxidative stress, especially when the protective role of DJ-1 has been compromised. If this was the case, then antisense inhibition of AMPK in DJ-1 knockdown strains should rescue the phenotypic defects observed after exposure to H_2_O_2_. This was found to be true, with knockdown of AMPK rescuing the phagocytosis, growth and fruiting body morphologies present after oxidative stress. Furthermore, the deranged phototaxis observed only in oxidatively stressed DJ-1 knockdown strains was rescued by antisense-inhibition of AMPK. This shows that these adverse phenotypic outcomes caused by oxidative stress are mediated by AMPK and exacerbated by the loss of DJ-1′s protective function.

To determine if DJ-1′s protective role was exerted at the level of preventing mitochondrial oxidative damage, respirometric analysis of mitochondrial function with and without oxidative stress was conducted in strains expressing altered levels of DJ-1 [46]. Basal OCR, maximum OCR, OCR consumption by ATP synthesis and OCR consumption by “proton leak” were all proportionately lowered by the exposure to H_2_O_2_ regardless of DJ-1 expression levels. Furthermore, elevated DJ-1 expression impaired mitochondrial respiration (rather than enhancing it) and DJ-1 knockdown enhanced mitochondrial function (rather than impairing it) in oxidatively stressed cells to the same extent as in unstressed cells [45]. Thus, although DJ-1 protects cells from the AMPK-dependent consequences of oxidative stress, it does not do so by protecting the mitochondria.

**Figure 1:**
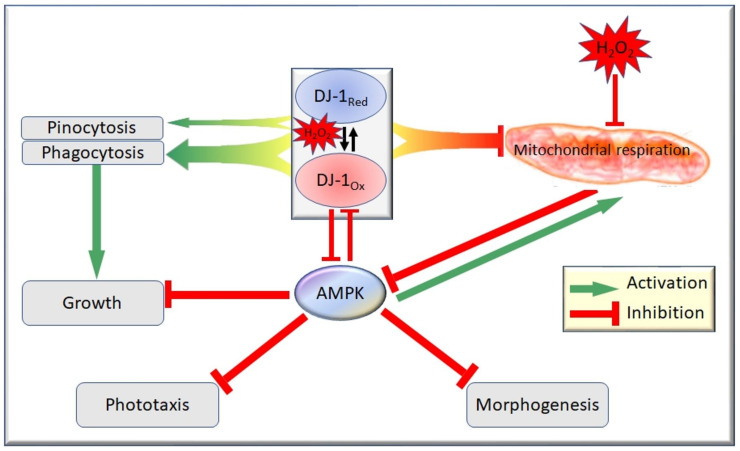
Model for phenotypic consequences of interactions between DJ-1, AMPK, mitochondrial respiration and oxidative stress. Mitochondrial respiration is inhibited separately by oxidative stress and DJ-1, both in its oxidized (DJ-1_Ox_) and reduced (DJ-1_Red_) states (indicated by the bifurcated base of the arrow). DJ-1 also activates phagocytosis (broad arrow) and to a slight extent (narrower arrow) pinocytosis, regardless of its oxidation state (indicated by the bifurcated base of the arrows). DJ-1_Ox_, but not DJ-1_Red_, inhibits and is inhibited by AMPK. Under oxidative stress mitochondrial respiration is inhibited, ATP production is compromised and AMPK is activated, by oxidative stress both directly (not shown) and via the impaired mitochondrial function. At the same time, DJ-1 is oxidized and inhibits AMPK, opposing the effects of impaired respiration and protecting cells from the downstream phenotypic consequences of chronic AMPK hyperactivity. Reproduced from Chen et al. 2021 [46].

*D. discoideum* has provided insight into the complexities surrounding DJ-1′s cellular roles in endocytic pathways and protection of cells during oxidative stress. The results were consistent with a model (Figure 1) in which DJ-1 and oxidative stress each exert independent inhibitory effects on mitochondrial respiratory function [46]. Under oxidative stress, AMPK becomes activated, and DJ-1 becomes oxidized. The chronic hyperactivity of AMPK has multiple cytopathological consequences, including impaired endocytosis and growth mediated via inhibition of oxidized DJ-1, as well as DJ-1-independent impairment of phototaxis, growth and morphogenesis. Genetic loss of DJ-1 function results in impaired endocytic pathways and consequently growth, these aberrant phenotypes being exacerbated by oxidative stress because of AMPK’s activation and the loss of DJ-1′s inhibition of AMPK under these conditions. The *D. discoideum* model has thus contributed to our understanding of DJ-1′s cellular roles and the mechanisms by which its loss can contribute to cytopathological outcomes, particularly in combination with elevated oxidative stress.

### 3.4. Alpha-Synuclein

*D. discoideum* has also allowed researchers the opportunity to study human PD-associated genes and genetic variants in the absence of endogenous genes and α-synuclein is one such example. Alpha-synuclein is involved in vesicle trafficking and is concentrated at the pre-synaptic terminals of neurons [69]. Misfolding of the protein results in a group of diseases named synucleinopathies which include PD as well as other neurodegenerative diseases such as multiple system atrophy (MSA) and dementia with Lewy bodies [70]. Misfolded α-synuclein is the most abundant protein in the characteristic intraneuronal aggregates or Lewy Bodies present in PD and is crucial to the progression of PD. Particular polymorphisms in the *SNCA* gene encoding α-synuclein substantially increase the risk of developing sporadic PD. Furthermore, multiplication and consequential overexpression of the *SNCA* locus, as well as certain point mutations within the gene result in early-onset, autosomal dominant PD [47]. Truncation of the C-terminus of α-synuclein increases the tendency of the protein to form cytotoxic oligomers as well as to aggregate [71]. However, the exact cytotoxic mechanisms underlying α-synuclein pathology remain elusive [13].

To create *D. discoideum* models for α-synuclein cytotoxicity, the wild type human protein and two PD-associated mutant forms of it were expressed in *D. discoideum* [13]. The full length (WT) and A53T mutated α-synuclein were found to be enriched in the cellular cortex, but the C-terminally truncated α-synuclein was found throughout the cytoplasm, suggesting the C-terminus is essential for cortical localisation. In support of this, a fusion protein consisting of the C-terminal 20 amino acids of α-synuclein fused to GFP localised to the cell cortex, demonstrating that these residues are not only necessary but are also sufficient for cortical localisation [13]. This result contrasts with reports of mitochondrial localisation of α-synuclein in human dopaminergic neurons [72,73] but agrees with other reports that α-synuclein is enriched in the cell cortex and its binding to membranes involves the N- and C-termini [74].

*D. discoideum* α-synuclein expressing strains were analysed for hallmark mitochondrial dysfunction phenotypes including defective slug phototaxis, reduced growth with unaffected endocytosis and impaired fruiting body morphology [13]. Strains expressing wild type α-synuclein displayed very mild phototactic defects which were not statistically significant, the A53T α-synuclein-expressing strains had a moderate defect and the C-terminally truncated α-synuclein-expressing strains showed a larger phototactic impairment in addition to impaired thermotaxis. Fruiting body morphology was not altered by the expression of WT or A53T mutated α-synuclein, however, C-terminally truncated α-synuclein, resulted in a reduced number of fruiting bodies which had thicker and shorter stalks. All species of α-synuclein caused defects in plaque expansion on bacterial lawns which was partially attributed to a reduction in phagocytosis [13].

With the exception of the defective fruiting body morphology observed in the C-terminally truncated α-synuclein strains, antisense inhibition of expression of the AMPK α subunit rescued or partially rescued all of these defective phenotypes [13]. This suggested that cellular stress, perhaps a reduction in ATP production, may be occurring in these strains and activating the energy sensor AMPK. The authors investigated mitochondrial function directly using Seahorse respirometry and showed that strains expressing α-synuclein did not have impaired but rather enhanced mitochondrial function [13]. Strains expressing the full-length wild type (WT) α-synuclein were most affected and displayed elevated basal and maximum respiration and the main components of these measures were also elevated. C-terminally truncated α-synuclein expressing strains also displayed these increases in respiration but only reached statistical significance for maximum respiration, not basal respiration. The smallest effect was seen in the A53T-expressing strains whose elevated measures did not reach statistical significance for any of the measured parameters. The increased respiration in the α-synuclein strains was not due to an impairment of any of the complexes, with the relative contributions of each complex to basal or maximum remaining unchanged. Therefore, the complexes were more active but functionally normal [13]. Whilst this elevated respiration is in contrast to some reports of mitochondrial dysfunction in PD, it accords with the elevated respiration rates in multiple cellular PD models [45,65,66,67].

The aggregation and accumulation of α-synuclein is thought to involve impaired interactions with other proteins and studying these interactions in model systems can assist in characterising the cytopathological disease pathways [13,14]. *D. discoideum* has been used to investigate the interaction of α-synuclein with the human microtubule-associated Tau protein [14]. *D. discoideum* lacks endogenous Tau and α-synuclein which allowed investigation of the effects of human Tau alone and in combination with human α-synuclein. The longest isoform of Tau was used to create a *D. discoideum* model. It was found that Tau was present throughout the cytosol where it was also seen colocalized in close association with tubulin [14]. In human cells, Tau is phosphorylated on many sites and in the phosphorylated state dissociates from microtubules [75]. *D. discoideum* was shown to phosphorylate Tau on at least one of these regulatory sites (S404) and to colocalise with tubulin in the cytosol. Strains expressing both α-synuclein and Tau showed that these two human neurodegeneration-associated proteins colocalised at the cortex where α-synuclein is most prominent. The close proximity (within 10 nm) suggests that Tau and α-synuclein have a physical interaction, not just a colocalization [14].

The strains expressing Tau or α-synuclein were examined phenotypically and shown to cause different patterns of phenotypic abnormalities [14]. Whilst expression of WT α-synuclein resulted in growth defects, impaired phagocytosis and increased mitochondrial respiratory activity, expression of Tau resulted in impaired phototaxis, thermotaxis, growth in liquid and an isolated respiratory complex V defect in oxidative phosphorylation. Tau expression did not alter plaque expansion rates on bacterial lawns, while α-synuclein expression did. Tau-expressing strains were also more susceptible to *Legionella sp.* infection, whereas α-synuclein strains were not. Tau-expressing strains displayed aberrant fruiting body morphology with shorter and thicker stalks, indicative of mitochondrial dysfunction, whereas α-synuclein expressing strains were comparable to WT.

When both proteins were coexpressed, a distinct profile of altered phenotypes was evident, different from that caused by expressing either of these proteins alone [14]. Thus, the coexpression of tau and α-synuclein is exacerbated (phototaxis, fruiting body morphology), or reversed (phagocytosis, growth on plates, mitochondrial respiratory function, *Legionella* proliferation) the abnormalities caused by either Tau or α-synuclein alone [14]. These results, together with the colocalisation experiments, indicate Tau and α-synuclein interact and influence each other’s cytotoxic effects.

To further elucidate the interaction between Tau and α-synuclein, whole-cell proteomic analysis was performed. Like the phenotypic data, the expression profiles of proteins in the Tau-expressing and α-synuclein-expressing strains were distinct. In Tau-expressing strains, cytosolic proteins were more abundant whereas, in α-synuclein expressing strains, the levels of cortex-associated proteins were elevated, consonant with the subcellular locations of both proteins. Genes associated with protein turnover were upregulated in strains expressing Tau, whereas strains expressing α-synuclein had no upregulation of genes involved in protein synthesis or degradation. This difference may reflect homeostatic feedbacks in Tau-expressing strains that increase protein catabolism as a means to increase ATP synthesis. In strains coexpressing both Tau and α-synuclein a downregulation of cytoskeletal genes was observed in all strain groups, which may explain the phenotypes observed such as impaired phototaxis, thermotaxis, phagocytosis and multicellular development, as these genes play key roles in these biological processes. The downregulated expression in these strains of proteins involved in cell morphogenesis, polarity and motility may explain why phototaxis was more deranged in these strains and “slugs” migrated shorter distances [14].

The results of ectopically expressing Tau and α-synuclein individually and in combination in the *D. discoideum* model have shown compelling evidence that these proteins interact in the cortex of cells, probably physically and also functionally in the altering of phenotypes. The phenotypic consequences highlight a complex relationship between the two proteins which could be further explored and produces distinct dysfunctional phenotypes suggesting that the two proteins exert their cytotoxic effects through distinct pathways and mechanisms [14].

Overall, *D. discoideum* has been used to investigate many of the genes which have been associated with PD [2]. These studies support recent data in multiple cellular models that mitochondria may not be impaired but hyperactive in PD, perhaps becoming damaged by elevated ROS production only in the later stages of the disease. Conversely, they also show that downregulation of AMPK in the PD models can rescue or partially rescue the disease phenotypes and suggests that AMPK may be hyperactive in PD [65]. This activation of AMPK is not anticipated to be due to altered AMP:ATP ratios, rather, it is most likely caused by other cellular stresses and the activation by upstream kinases such as CaMKKβ. An influx of cellular calcium, also implicated in PD, can result in the activation of AMPK via CaMKKβ [76]. The relationship between PD genes, calcium dysregulation and AMPK activation could easily be investigated in *D. discoideum*. Several models of PD have been created in *D. discoideum* and future work using these models may further characterise what the normal roles of the associated proteins are in healthy cells and how mutations can result in altered signalling pathways. Since the key pathways implicated in PD (mitochondrial and lysosomal pathways) are well studied and characterised in *D. discoideum*, future research is likely to assist in our understanding of these processes in PD cytopathology.

## 4. Huntington’s Disease

Huntington’s disease (HD) is a fatal neurodegenerative disease that is characterised by involuntary movements, cognitive decline and psychiatric symptoms. The disease presents clinically in the 4th and 5th decades of life and the average life expectancy is within 15–20 years of disease onset. HD is an autosomal dominant disorder caused by mutations in the huntingtin (*htt*) gene. The mutations result in an expansion of the CAG trinucleotide repeat in excess of 35 repeats. The normal and mutant Huntingtin protein is expressed ubiquitously but certain neuronal populations are more susceptible to damage and death, specifically striatal and cortical neurons [77]. In humans, numerous roles have been ascribed to the Huntingtin protein including roles in embryogenesis and development, apoptosis, autophagy, transcription, vesicle trafficking, axonal transport and mitochondrial impairment. It is widely accepted that the mutation results in a gain of function, but it may also interfere with Huntingtin’s normal role [78].

The Huntingtin protein has been well conserved in evolution and is present in all eukaryotes with the exception of plants and fungi. *D. discoideum* contains a single *htt* gene (DDB0238473) encoding the Huntingtin homologue, Htt. The protein contains a polyglutamine tract which is 19 residues in length, comparable to the normal range of the expansion in humans. The polyglutamine residues are encoded by the CAA codon interrupted by a single CAG codon and are not positioned in exon 1 as in mammals [79]. Santarriaga et al. [80] genetically manipulated the *D. discoideum* Htt protein by creating a polyglutamine expansion in exon 1 and showed that unlike all other organisms tested including yeast and mammalian cells this protein did not aggregate. *D. discoideum* encodes a large number of proteins with polyglutamine expansions and such proteins in other organisms including mammals are generally associated with protein aggregation and disease, yet in *D. discoideum* these proteins do not aggregate. The properties that make *D. discoideum* proteins with polyglutamine stretches resistant to aggregation are not clearly defined but it has been suggested that chaperone proteins specifically small heat shock proteins may be involved and *D. discoideum* encodes a large number of these proteins [80]. The Htt protein is expressed throughout development and is located in the cytosol [79]. *D. discoideum htt* gene knockouts have been created to study the function of the protein and, as in mammalian cells, *D. discoideum* Htt is multifunctional.

Htt in *D. discoideum* functions in growth and development, but unlike in higher eukaryotes where it is embryonically lethal, *D. discoideum* Htt^−^ mutants are viable. When Htt^−^ cells were grown in adherent culture their growth rate was unimpaired [79] but when grown in an axenic shaking culture they grew slower than the wild type parental strains [81]. This difference in ability to grow in different media was found to be due to a defect in the EDTA-resistant cell adhesion properties in shaking culture. The Htt null cells showed an altered expression of the key regulator of EDTA-resistant cell-cell adhesion contact site A (csA) glycoprotein. These altered-expression and EDTA-resistant cell adhesion properties were rescued in the null mutant by supplementation with calcium [79,82]. It is unknown how calcium can rescue these defects, but the results clearly show that Htt plays a role in cell adhesion in *D. discoideum* as it does in higher eukaryotes suggesting this function is well conserved through evolution. *D. discoideum* provides a useful system to further interrogate this pathway and determine how the repeat expansion interferes with this function.

HTT, the human Huntingtin gene, is essential and its deletion is embryonically lethal in mice [83]. In *D. discoideum,* the Htt protein also plays a role in development with Htt^−^ mutants showing delayed development, beginning with a delayed onset of aggregation and streaming and failure of many mounds to progress through further development. The fruiting bodies which did form did so at a delayed rate and the sori had a glassy appearance, due to the premature germination of spores in the sori with a decrease in spores with increasing age of the fruiting bodies. Spores were also less viable and less efficiently formed [79]. Using chimeras, Myre et al. [79] showed that *htt* null cells in the presence of wild type cells would not populate the prespore region and consequently did not end up in the spores. Furthermore, Bhadoriya et al. [81], using LacZ reporter fusion and neutral red staining demonstrated a misregulation of spatial expression of prestalk cells. Conversely, the expression of prespore cells was the same as the wild type until it reached the slug stage and its expression significantly increased in the *htt* mutant. The authors then examined the expression of STAT transcription factors and noted a decrease in the expression of STATs involved in the decision to proceed with slug migration or culminate. Together this data shows that Htt is required for proper patterning and maintenance of stalk and spore cell boundaries [81].

The delay in aggregation that was observed in the Htt^−^ cells was due to a defect in cAMP signalling. The ability of starving cells to aggregate under conditions of nutrient stress is dependent on chemotaxis to cAMP. Thompson et al. [84] showed that the speed and accuracy of chemotaxis is reduced in the *htt*^−^ mutant [84]. Bhadoriya et al. [81] analysed cAMP signalling in *htt*^−^ cells by measuring cAMP protein levels and levels of transcripts of key genes. They noted a reduction in absolute cAMP levels during all stages of development with no characteristic increases during starvation or in the loose aggregate stage. cAMP is produced by three different adenylate cyclases and all three showed altered levels of transcripts in the htt^−^ cells. AcA (adenylyl cyclase of aggregation) which is normally expressed during aggregation was reduced at all developmental stages, AcrA (adenylyl cyclase with response regulator domain) normally expressed at mid development and is essential for spore production was reduced in later stages of development and AcgA (adenylyl cyclase of germination) which is important for maintaining spore dormancy was increased during development [81]. Furthermore, the catalytic subunit of protein kinase A (PKA) was increased in the vegetative cells, but was reduced in the slug and fruiting body whereas the regulatory subunit was decreased at all stages of development. This suggests that PKA in *htt*^−^ cells is regulated independently of cAMP and ensures that culmination can proceed despite the reduced cAMP signalling and delayed aggregation [81].

Myosin II forms filaments in chemotaxis and is required for the structural reorganisation of the actin network and contraction of the posterior of the cell. It normally accumulates in the cortex after cAMP stimulation and the amount of myosin II decreases in wild type cells after stimulation with cAMP [85]. This did not occur in the *htt*^−^ mutants which did not show a cAMP-stimulated accumulation in the cortex, displaying a reduced amount of myosin II in the triton-insoluble fraction that did not change with cAMP signalling. Myosin II is regulated by its phosphorylation status and the amount of phosphorylated myosin II was increased in the *htt^−^* mutant [85].

Myosin II is also required for cytokinesis where it accumulates at the cleavage furrow. In agreement with their altered myosin II function, the *htt*^−^ mutant cells displayed a defect in the late stage of cytokinesis on non-adherent coverslips. Myosin II accumulated at the cleavage furrow early in cytokinesis but was subsequently lost from this location, likely due to a reduction in the activity of PP2A and an increase in phosphorylated myosin II [85].

Myosin interacts with the actin cytoskeleton whose complex rearrangements are necessary for normal motility and division. In addition to the cytokinesis and chemotaxis defects, Htt-vegetative cells displayed defects in response to changing environmental conditions. When Htt^−^ cells were grown in a non-nutrient buffer a reduction in pseudopods and a rounding of the cells was observed. This was accompanied by a relocation of F-actin from the cortex to the cytosol. The *htt*^−^ cells were also sensitive to osmotic shock and, unlike wild type cells, when placed in water did not form contractile vacuoles, lost adhesion and lysed. Thus, the *htt*^−^ cells have a defect in contractile vacuole activity under hypotonic stress [79].

As in other organisms Htt in *D. discoideum* is multifunctional and many of these functions seem to be conserved across evolution. Further characterising these roles in *D. discoideum,* where genetic manipulation of the pathways is possible and multiple phenotypes are measurable, will increase our understanding of the normal role of Htt and how mutation of the protein can lead to Huntington’s Disease.

## 5. Neuronal Ceroid Lipofuscinoses

Neuronal ceroid lipofuscinoses (NCLs), together referred to as Batten disease, are rare, inherited neurodegenerative disorders [86]. NCLs typically affect children, however, onset can occur in adulthood [87] Common symptoms of NCLs include dementia, vision loss, epileptic seizures, and premature death [88]. The disease is characterised by the accumulation of autofluorescent storage material called lipofuscin in neuronal and peripheral cells and the death of neurons in the retina and brain [88,89]. There are 13 *CLN* (ceroid lipofuscinoses, neuronal) genes and over 430 autosomal recessive and dominant mutations within these genes have been associated with NCLs [89]. The *CLN* genes encode proteins that are predominantly associated with lysosomes, endosomes, and the endoplasmic reticulum [89]. Due to this, NCLs are often referred to as lysosomal storage disorders. NCL disorders can be classified into subtypes based on the *CLN* gene mutated and the age of onset. The *CLN* genes encode several different proteins—soluble lysosomal enzymes/proteins (CLN1, CLN2, CLN10 and CLN13), transmembrane proteins (CLN3, CLN6, CLN7, CLN8, CLN12), a secretory pathway protein (CLN11), and peripherally associated membrane proteins (CLN4 and CLN14) [90]. The exact function and role of each of the CLNs in Batten disease is not well understood. *D. discoideum* provides an excellent model system for studying NCLs as there are 11 homologues of the 13 associated genes (see Table 2) [8]. Although *D. discoideum* does not contain homologues of the ER-membrane proteins CLN6 and CLN8 [91], it is a well-established model for studying the endocytic and lysosomal signalling pathways implicated in NCL. Human genes have also been shown to rescue *D. discoideum* NCL-associated knockouts, indicating conserved roles for the proteins in *D. discoideum* [92]. The following sections will describe the characterisation of *D. discoideum* NCL-associated homologues and highlight how the model organism has contributed to the understanding of NCLs.

### 5.1. CLN1/PPT1

In humans, the *CLN1* gene encodes the lysosomal enzyme palmitoyl-protein thioesterase 1 (PPT1) [118,119]. Ablation of PPT1′s enzyme activity results in infantile NCL and a reduction of the activity causes juvenile NCL with individuals living to early adulthood [120,121]. PPT1 removes long-chain fatty acids from modified cysteine residues and therefore is responsible for the reversal of a post-translational modification termed palmitoylation [119]. A reduction of PPT1 activity then leads to a build-up of thioesters as has been demonstrated in the mouse and lymphoblast models. PPT1 is secreted and contains a signal peptide for this which is conserved in the *D. discoideum* homologue [91]. *Ppt1* in *D. discoideum* encodes the protein (Ppt1) which, like most other CLNs, is part of the macropinocytosis pathway. PPT1 is believed to regulate phagocytosis, in line with its postulated roles in other model systems [91]. For example, in the mouse PPT1 null mutant, phagocytic cells were shown to be rapidly recruited to the brain following apoptosis of neurons causing loss of viable neurons and increasing NCL progression [91]. Studying Ppt1 in *D. discoideum* may elucidate its precise role in the endocytic pathway and how it regulates phagocytosis. This may in turn shed light on how the loss of PPT1′s enzyme activity in humans leads to the progression of CLN1 disease.

### 5.2. CLN2/TPP1

In humans, the *CLN2* gene encodes the inactive lysosomal aminopeptidase tripeptidyl peptidase I (TPP1) [122,123]. Mutations in the *CLN2* gene typically result in late-infantile NCL whereby children will present with symptoms around the age of two and usually die between the ages of 6.5–12.5 years old [124]. Later-onset NCL is also caused by *CLN2* mutations, whereby disease progression is slower and clinical symptoms appear around 5–7 years of age [125]. Children with later-onset CLN2 will succumb to the disease in their teenage years [126]. Cerliponase alfa (Brineura™), a TPP1 enzyme replacement therapy, is currently used to delay disease progression [127].

Upon translocation and acidification of inactive TPP1, autoproteolysis of the protein results in an active, enzyme localised to the lysosome [128,129]. It functions as a protease and cleaves N-terminal tripeptides from substrates in addition to being a weak endopeptidase [130,131]. The storage material which accumulates in neurons, due to the loss of TPP1 activity, mainly consists of subunit c of mitochondrial ATP synthase (SCMAS) [132]. TPP1 protease activity appears elevated when Batten disease is caused by mutations in *CLN3* indicating a common biological pathway for CLN2 and CLN3 disease [133]. The physiological role of TPP1 in humans remains unclear. The protein is widely conserved in vertebrate models but lacks homologues in several commonly used simple eukaryotic models such as *Caenorhabditis elegans*, *Saccharomyces cerevisiae* and *Drosophila melangoster* [96].

*D. discoideum* on the other hand has six homologues of TTP1, three of which are more structurally similar to the human homologue (Tpp1A-C) [17]. Tpp1D-F all contain an additional, conserved, peptidase domain. *Tpp1A* encodes a protein, localised to the lysosome, and was the first homologue characterised in *D. discoideum* [91]. In nutrient-rich conditions expression of Tpp1A in vegetative cells is relatively low [8]. Upon starvation, expression increases significantly, peaks midway through multicellular development and begins to decline. Loss of Tpp1A in *D. discoideum* results in the accumulation of autofluorescent storage material typical of Batten’s disease [96]. The null strain also limits spore formation and is believed to play a role in cell differentiation [96]. Tpp1A appears to regulate autophagy and cells become less viable in autophagic inducing media if Tpp1A is reduced. These phenotypes support other models which have shown a role for CLN2 in autophagy [134].

Knockdown strains revealed that the reduction in Tpp1A expression correlates with smaller slugs which developed into smaller fruiting bodies [17]. Despite the effect of loss of Tpp1A on slug size, the accuracy of phototaxis remained unaffected. Tpp1A knockdown also resulted in reduced growth rates on bacterial lawns and in liquid medium. Interestingly, despite the slow growth, endocytosis (phagocytosis and macropinocytosis) was elevated when Tpp1A levels were reduced. This was postulated to be due to altered TOR (Target of Rapamycin) signalling and some additional experiments supported this [17]. The authors inhibited TOR pharmacologically with Rapamycin treatment and genetically with antisense inhibition of Rheb expression. Rheb (Ras homolog enriched in brain) is a small monomeric GTPase which is highly conserved amongst eukaryotic organisms. It is a positive regulator of TOR signalling and in its GTP-bound form activates TOR. In both cases, the resultant strains phenocopied knockdown of Tpp1A. To further validate the finding that Tpp1A was a regulator of TOR, cotransformants were created in which Rheb was overexpressed and Tpp1A expression was antisense-inhibited to determine if Rheb could rescue defects seen in Tpp1A-knockdown strains. An increase of Rheb protein expression in cells with decreased Tpp1A resulted in strains phenotypically akin to WT [17].

Restriction enzyme-mediated integration (REMI) mutagenesis was employed in *D. discoideum* null strains and a secondary suppressor of Tpp1A subsequently named Suppressor of Tpp1A (StpA) was identified [96]. This suggests the need to determine if humans have second site suppressors of TPP1 which could be a target for therapeutic development. Following the initial characterisation of Tpp1A in *D. discoideum* further TPP1 homologues were identified, two of which have been investigated [17].

*Tpp1B* and *tpp1F* [8] are both involved in macropinocytosis and are highly expressed in vegetative cells. Following starvation and the induction of multicellular development, the expression of both proteins is reduced, in contrast with the expression of Tpp1A, whose expression is highest during multicellular development and lowest during cell growth. This suggests that each Tpp1 protein has different functions or roles within the cell at least in terms of development.

A recent study revealed for the first time that Tpp1B and 1F interact with a Golgi membrane channel called Golgi pH regulator (GPHR) [98]. This channel is ubiquitous in eukaryotes and its function is to acidify compartments of the Golgi complex [135]. The interactions between GPHR and Tpp1 homologues warrants investigation in higher organisms to determine its relevance to humans and CLN2 disease. Tpp1B and Tpp1F bind the GPHR via its DUF3735 domain [135]. Mutants have been created in *D. discoideum* which lack the GPHR protein, and these mutants display membrane trafficking, growth, and developmental defects [135]. Cells lacking Tpp1F have been investigated and they show no defects in growth or development which may be due to compensation by Tpp1B [135]. Creation of Tpp1B null mutants and TPP1B/IF double mutants would be useful to answer this question. In addition, it would also be valuable to generate and characterise knockouts of the remaining *Tpp1* genes in *D. discoideum*.

### 5.3. CLN3

*CLN3* encodes a lysosomal/endosomal transmembrane protein, Battenin [73,136]. CLN3 disease is the most prevalent form of NCL and over 50 NCL-associated mutations have been detected in the *CLN3* gene [136]. Approximately 80–90% of CLN3 patients have a 1.02 kB deletion resulting in truncated Battenin. This causes juvenile CLN3 disease, where clinical presentation can occur between the ages of 4–7 years, and patients usually succumb to the disease between the ages of 16–35 years [137]. Battenin has been shown to participate in several different cellular processes including, but not limited to synaptic transmission, autophagy, apoptosis, endocytosis, ion homeostasis and cell cycle control [138,139,140,141,142] Dysregulation of calcium has been implicated in synaptic dysfunction and premature apoptosis in neuronal cells of patients with CLN3 disease [92]. Out of all the biological functions Battenin is involved in, its role in the lysosome has been the most extensively studied. Despite this, the precise normal physiological role of Battenin and its role in CLN3 disease is still unknown [143].

*Cln3* in *D. discoideum* encodes the protein Cln3 which is primarily localised to contractile vacuoles (CV) and is postulated to play a role in maintaining cellular osmolarity [91,100]. *D. discoideum Cln3^−^* vegetative cells were less resistant to osmotic stress with a reduction in viability as determined by a reduction in ATP levels after exposure to a hypotonic solution [100]. In addition, spores had less integrity and were more likely to germinate unexpectantly during osmotic stress when compared to WT. This is in line with mammalian CLN3 disease models which show defects in osmoregulation due to the loss of CLN3 [144]. *Cln3^−^* cells display defects in cytokinesis believed to be due to dysregulation in osmolarity, as CV’s are crucial to cleavage furrow formation [100].

In addition to defects in osmolarity and cytokinesis, loss of Cln3 resulted in the accumulation of storage material, loss of adhesion and an increase in cell proliferation accompanied by reduced expression of autocrine proliferation repressor, AprA [92]. Developmental time-course experiments revealed that the lack of Cln3 accelerated each phase of the *D. discoideum* multicellular life cycle. When *Cln3^−^* cells were transformed to express human CLN3 or *D. discoideum* Cln3, the subsequent timing of each phase of the developmental cycle was rectified and aberrant slug migration was also restored. These phenotypes were also rescued using the calcium chelator EGTA, further validating CLN3′s potential role in calcium regulation. RNAseq studies of *Cln3^−^* cells revealed that during hypotonic stress the CLN2 homologue, Tpp1A, was upregulated and its activity was increased, demonstrating an interaction between CLN proteins in *D. discoideum* which could also occur in humans [102].

CVs are also thought to be involved in the unconventional secretion of proteins that lack secretion peptides [103]. Cln3 in *D. discoideum* was found not only localised to CVs but also the Golgi complex where proteins with secretion peptides are transported, suggesting an important role for Cln3 in both conventional and unconventional protein secretion. Mass spectrometry analysis was used to detect secreted proteins in *cln3^−^* conditioned media and revealed a reduction in the number of secreted proteins. Two adhesion proteins were absent, possibly explaining why *Cln3^−^* cells display aberrant cell adhesion. Along with limited secretion of cell adhesion proteins, there was also a reduction in secretion into the medium of countin, a proliferation repressor protein, and this may explain the increase in cell proliferation in *cln3^−^* cells [103]. The reduction of protein secretion in *cln3^−^* cells needs to be explored further and may include contributions from improper protein expression and turnover. Proteomic analysis of a CLN3 knock-in mouse model also revealed that CLN3 is crucial to the formation and function of lysosomes and the lack of CLN3 may result in reduced protein production and thus secretion [145].

In addition to altered secretion of proteins, altered expression of other *D. discoideum* NCL homologues including Tpp1F, CtsD and Cln5 has been observed in *cln3*^−^ cells [103]. Cln5 was of particular interest as its secretion was found to be elevated in *cln3^−^* cells and it was subsequently revealed to colocalise with Cln3 in CVs [8,103]. RNAseq analysis has also been performed on *D. discoideum* *cln3^−^* cells during growth, starvation and osmotic stress and revealed increased expression of TPP1A in *cln3^−^* cells under starvation and osmotically stressed conditions, but not during growth [102]. This provided more evidence that NCL proteins interact and may do so in humans either directly or through intermediates. The analysis also revealed reduced lysosomal enzymes during starvation in *cln3^−^* cells which has been noted in post-mortem brains of NCL patients [102].

### 5.4. CLN4/DNAJC5

CLN4 disease results in a rare adult NCL which typically begins between the ages of 25–46 years and triggers early-onset dementia and movement disorder, however, does not cause blindness like most other NCLs [146]. CLN4 disease is caused by mutations in the *DNAJC5* gene, resulting in the aberrant production of cysteine string protein alpha (CSPα) [147]. CSPα, is essential to propagating nerve impulses between neurons by recycling the misfolded proteins involved [148,149]. It is unclear how mutations in *DNAJC5* result in the accumulation of storage material [150]. A true homologue of CLN4 is yet to be identified in *D. discoideum*, but two genes have been identified to show significant similarity [8,91]. *D. discoideum* DnaJ homolog 1 (Ddj1) is the least likely to be a homologue of *DNAJC5* as the protein produced is significantly larger than CSPα. *DDB_90290017* which encodes an uncharacterised protein appears to be the more likely candidate [8].

### 5.5. CLN5

Mutations in *CLN5*, cause variant late-infantile NCL [151]. CLN5 is poorly understood, but studies have revealed mature forms of the protein are localised to the extracellular space and lysosome. CLN5 has been found to interact with several other NCL proteins and has been linked to apoptosis, autophagy, cell growth, myelination and intracellular trafficking, sphingolipid transport and synthesis [151]. CLN5 homologues are absent in many lower eukaryotic models but *D. discoideum* encodes a Cln5 homologue whose loss results in the accumulation of storage material, the hallmark of NCL disease [107]. *D. discoideum* Cln5 is post-translationally modified by glycosylation in the ER, trafficked to the cortex and secreted during multicellular development [107]. Appropriate glycosylation is essential to the folding and trafficking of CLN5 to the lysosome in humans and aberrant glycosylation in CLN5 disease is believed to cause the retention of the protein in the ER [152]. Modifying glycosylation sites on *D. discoideum* Cln5 may provide insight into the inappropriate trafficking of CLN5 when mutated.

*D. discoideum* secretion of Cln5 was impaired following treatment with lysosomotropic compounds implicating a role for autophagy in the regulation and secretion of the protein [107]. Loss of Cln5 results in defective cell–cell and cell–substrate adhesion, in nutrient-depleted cells which is exacerbated when lysosomal pH is increased further implicating Cln5′s link to autophagic mechanisms [107]. Poor cell-substrate adhesion has also been observed in fibroblasts from NCL patients with a CLN5 mutation [107]. Chemotaxis, most likely due to the depleted cell adhesion, is also reduced in *cln5*^−^ cells [107]. The *cln5*^−^ cells exhibited a limited chemotactic response to folic acid and accelerated progression through the multicellular life cycle [153]. Research initially using *D. discoideum* has revealed that both *D. discoideum* Cln5 and human CLN5 function as a glycoside hydrolase [101]. This knowledge may be used to design enzyme replacement therapy, as similar therapies have been successful in delaying the progression of CLN2 disease due to loss of TPP1 activity.

### 5.6. CLN7/MFSD8

In humans, *CLN7* encodes a lysosomal protein named MFSD8 (major facilitator superfamily domain-containing 8) [154,155]. Mutations in *MFSD8* cause variant late-infantile NCL disease whereby onset of clinical symptoms occurs between 2–7 years old, and children usually live until late childhood or early teenage years. MFSD8 localises to the lysosomal membrane and is thought to be involved in the transportation of substances across cellular membranes [156].

In *D. discoideum,* the *mfsd8* gene encodes the protein Mfsd8 which localises to endocytic compartments [108]. Expression of the protein remains relatively stable during development, however, increases 8 h after starvation suggesting a role for Mfsd8 in chemotaxis (cAMP mediated) and aggregation [108]. When pulled down, mass spectrometry revealed, Mfsd8 interacts with other NCL proteins such as CtsD and proteins associated with Cln3 and Cln5 [108]. So far, these interactions have only been described in *D. discoideum,* however, the roles of Mfsd8 may be conserved and warrant further investigation in higher eukaryotes.

### 5.7. CLN10/CTSD

The human *CTSD* gene encodes Cathepsin D (CTSD) and mutations in this gene result in CLN10 disease [157]. Seizures are a predominant indicator of congenital CLN10 and can occur in utero then often fail to respond to treatment after birth. Symptoms are apparent following birth and include respiratory failure, muscle stiffness and microcephaly [158]. Sadly, these infants tend not to survive the first hours or weeks following birth [159]. Late-infantile CLN10 also results in seizures and problems with vision, ataxia and impaired intellectual skills. Children with late-infantile CLN10 often do not survive past early childhood [158]. Cathepsin D is a vital lysosomal proteinase expressed in most tissues and a decrease or lack of production of the protein results in widespread neuronal loss [160]. In humans, CTSD is proposed to be involved in neuronal polarity and migration. and to play a role in oxidative and staurosporine-induced death in human fibroblasts and cancer cells, respectively [160]. In *D. discoideum*, *ctsD* encodes a protein containing a secretion signalling peptide and localises to endocytic compartments [91]. CtsD is more highly expressed during growth and aggregation and when CtsD was depleted aggregation was delayed in *D. discoideum.* Therefore, the role of *D. discoideum* CtsD is comparable to the known roles in humans [8].

### 5.8. CLN11/GRN

The human *GRN* gene produces a protein called progranulin, a lysosomal membrane protein, which is subsequently cleaved by lysosomes into granulins [161]. Mutations to GRN result in an autosomal recessive form of NCL disease [91] characterised by epilepsy, retinal dystrophy, cerebellar ataxia and cognitive decline in adolescence or early adulthood [162]. These mutations cause disruptions to the production of granulins. Little is known about the protein’s role within the brain and its study in a simple model system could provide insight into the normal and pathological roles of granulins [8,163]. *D. discoideum* could provide such a system, as its genome encodes a clear homologue (Grn) [97].

### 5.9. CLN12/Park9

Mutations in *CLN12 (Park9)*, encoding the protein ATP13A2, result in juvenile-onset NCL in addition to Parkinson’s Disease type 9 (also referred to as Kufor-Rakeb syndrome) [164,165]. ATP13A2 is a P-type endolysosomal cation-transporting ATPase which is believed to facilitate uptake of polyamine and protect cells against manganese and nickel toxicity [164,166]. *D. discoideum* encodes a homolog of human ATP13A2 named *kil2.* Kil2 acts as a magnesium pump in phagosomes to aid in the efficient killing of bacteria. Furthermore, Kil2 null mutants show aberrant cation stress.

### 5.10. CLN13/CTSF

The human *CTSF* gene encodes Cathepsin F (CTSF) [167]. CLN13 disease, caused by autosomal recessive mutations in the *CTSF* gene, results in adult-onset NCL. The disorder, also known as Kuf’s type B disease, is characterised by a progressive cognitive decline leading to dementia and cerebellar atrophy with patients sometimes developing seizures. CTSF is a soluble lysosomal cysteine protease associated with autophagy, cell immunity, lipoprotein and proteasome degradation [90]. It is unclear how mutations in the *CTSF* gene cause CLN13 disease, however, the progression of the disease is notably slow compared to other NCLs suggesting CTSF’s role is somewhat compensated [90].

In *D. discoideum*, the probable homologue of CTSF is cysteine protease A (CprA), but additional proteins show sequence similarities including cysteine protease B (CprB) and the uncharacterised protein DDB0252831 [8]. DDB0252831 is expressed during growth whereas both CprA and CprB are only expressed during development following cAMP signalling. All *D. discoideum* CTSF homologues, like human CTSF, can be secreted and in *D. discoideum* this has been shown to occur during starvation. All three candidates would be useful to study in *D. discoideum* to identify the functional homolog(s) of CTSF [8].

### 5.11. CLN14/KCTD7

CLN14 disease is an autosomal recessive disorder resulting in infantile NCL and caused by mutations in the *KCTD7* gene [168]. The gene encodes a highly conserved protein named potassium channel tetramerization domain containing 7 (KCTD7) [169]. Mutations to KCTD7 were commonly associated with myoclonic epilepsy but have now been shown to cause NCL. Both diseases result in epilepsy, but CLN14 disease is distinguished by the classical NCL accumulation of storage material along with motor, speech and visual impairment [168]. There are four potential homologues of KCTD7 in *D. discoideum*, Kctd9 and three uncharacterised proteins (DDB0238663, DDB0346929, and DDB0347398) [8]. Establishing which homologue is functionally similar to KCTD7 would allow for a *D. discoideum* model to be developed for CLN14 disease.

Ultimately, the presence of many homologues of NCL-associated proteins in *D. discoideum* provides an excellent opportunity to study their role in conserved pathways and how they interact with each other. *D. discoideum* is renowned for its genetic tractability and is commonly used to investigate how certain mutations lead to disease. Many NCL models have now been established in *D. discoideum* and other simple eukaryotic model systems contributing to the understanding of this group of disorders [91].

## 6. Lissencephaly

Lissencephaly, a severe developmental disorder affecting human infants is characterised by the loss of folds in the cerebral cortex. Whilst its incidence is rare (1:100,000 births), the prognosis is often devastating, with most children succumbing to the disease before the age of 10 [170,171]. The severity and symptoms associated with the disease vary between individuals [172]. Symptoms may include microcephaly, difficulty swallowing, muscle spasms and impaired development [173]. Lissencephaly is caused by mutations in cytoskeletal genes such as *PAFAHB1* and *DCX*. *PAFAHB1* [174] encodes the LIS1 protein. LIS1 is a key regulator of dynein-mediated transport along microtubules within cells and is a crucial protein to neurodevelopment [175]. The loss of folds or smooth appearance of the brain is believed to result from impaired migration of neurons during development [176]. Mutations in the *DCX* gene, which encodes the microtubule-associated protein doublecortin, also cause lissencephaly, however, these mutations are much less common [174]. Mutations in actin cytoskeleton-regulating proteins such as filamin have also been shown to cause a milder form of lissencephaly, periventricular heterotopia, but these will not be discussed here [177].

*D. discoideum* contains a homologue of both LIS1 and DCX [177]. Studies performed on *D. discoideum* Lis1 provided the first evidence of a direct interaction of the protein with regulators of actin. Lis1 was shown to interact with Rac1A, which aids in the reorganisation of the actin cytoskeleton of *D. discoideum* [178]. This interaction indicated that Lis1 not only played a role in microtubule organisation but also aided in actin regulation.

To further investigate Lis1′s role in actin and microtubule organisation in *D. discoideum* Lis1 mutants were isolated [178]. A Lis1 *D. discoideum* knockout was not achievable possibly due to its lethality, but a Lis1-D327H point mutant was created corresponding to the human D317H point mutation implicated in several lissencephaly cases. Firstly, localisation experiments found that wild type Lis1 was concentrated on the microtubule network and at the centrosome. Further analysis revealed that Lis1 localised to the centrosome independent of microtubules. The localisation of Lis1-D327H was indistinguishable from wild type Lis1, but the Lis1 mutants showed altered actin dynamics and the Golgi apparatus appeared dispersed. Complete disorganisation of the microtubule network was apparent with microtubules no longer interacting with the cortex and appearing to be dragged along by faster-moving centrosomes in the cytosol [178]. Disruption of the microtubule network was also noted in cells overexpressing wild type Lis1, however, the phenotype was considerably less drastic [178]. In *D. discoideum* centrosomes are cytosolic but remain closely associated with the nucleus, Lis1-D327H point mutant cells showed a decreased association of centrosomes with the nucleus. The consequences of loss of function of Lis1 could be attributed to perturbed dynein/dynactin regulation [178].

As aforementioned, *D. discoideum* has a conserved homologue of doublecortin, which is implicated in X-linked lissencephaly due to mutations in the *DCX* gene. In humans, doublecortin is thought to play a critical role in neuronal migration during development. It appears to play a similar role in *D. discoideum*, whereby it is only expressed during multicellular development [177]. Unlike in humans, doublecortin is not critical to cell migration and when knocked out, no observable defects in cell migration during photo/chemotaxis were observed [177]. No defects in migration were observed for Lis1-D327H point mutant cells either [177]. Loss of doublecortin activity seems to be compensated by Lis1, as cells carrying the Lis1-D327H point mutation failed to stream and aggregate effectively when doublecortin was also knocked out [177].

*D. discoideum* provides the only lower eukaryotic model with homologues of all the pertinent lissencephaly proteins. It has provided an excellent simple model for investigating defects in actin and microtubule organisation and elucidating interactions between lissencephaly-associated proteins with dynein and cytoskeletal proteins. It has provided evidence that Lis-1 regulation of actin may be important to neuronal motility which is disrupted in lissencephaly. Further studies using *D. discoideum* may provide a clearer molecular insight into lissencephaly in infants [11].

## 7. *D. discoideum* as a Pharmacological Model for Neurological Disorders

Many medicines and traditional or herbal remedies associated with health or medicinal benefits have been shown to be effective in clinical trials, but the underlying mechanism(s) of action or direct targets remain to be confirmed. Defining these targets is particularly difficult in mammalian models due to the genetic redundancy of higher organisms and the absence of genome-wide screening approaches for identifying direct molecular targets.

*D. discoideum* offers an innovative model for pharmacological research in the identification of roles for gene/proteins in disease pathways or potential pharmaceutical targets [179]. The compact haploid genome of *D. discoideum*, with a reduced number of protein orthologues compared to mammalian models, and single or multiple genes that can be easily ablated using CRISPR/Cas9 technology in the same cell line [180], provides a more simple system for identifying evolutionarily conserved molecular targets for bioactive compounds. This reduced complexity also simplifies various ‘omics’ approaches, including the analysis of bioinformatics analysis of RNA expression (RNAseq [181]), or proteomic data sets. In addition, the distinct phases or unicellular proliferation and multicellular development enable the identification of distinct effects of compounds on the two life cycle stages, in addition to distinguishing effects of the generalised cell toxicity in blocking both cell proliferation and development. Unlike some model systems, multiple gram weights of isogenic cell lines can be easily prepared for biochemical analysis, including cells having lost a target protein, or expressing a mutated form of a *D. discoideum* or human protein [27,28]. However, most importantly, libraries of insertional mutants can be rapidly employed to identify compound-resistant mutants [182] including the recently developed REMIseq [182,183], which can be rapidly recapitulated using CRISPR technology [180]. These approaches can also be used in the identification of suppressor mutants, by either random mutation or by targeting candidates [184]. Thus *D. discoideum* offers a range of advantageous attributes and experimental approaches to identify potential primary target proteins for poorly characterised drugs or bioactive compounds.

There are several key steps to the use of *D. discoideum* as a pharmacological model. The first is that cell proliferation must be reduced by the compound at clinically relevant concentrations or used in previous in vitro studies. This is important to reduce the likelihood of identifying off-target proteins or signaling pathways related to the side effects of the compound. Many *D. discoideum* studies have employed a wide range of drugs and bioactive natural produces at relevant concentrations to identify compound-resistant mutants. These compounds include cannabis-derived medicines for the treatment of epilepsy and multiple sclerosis [185,186], flavonoids and related compounds for the treatment of polycystic kidney disease and Alzheimer’s disease [187,188], fatty acids associated with epilepsy or tuberous sclerosis treatment [189] and drugs used in the treatment of bipolar disorder [190,191]. Secondly, this approach is likely to identify a range of potential protein targets, and care must be taken when selecting targets for further analysis. The potential target protein must have close orthologues in humans (in medicinal studies), and evidence for the protein or related signalling pathway as a target or in related disease function will increase the potential for successful translation. The subsequent characterisation of the target protein must include the deletion of the gene in multiple independent mutants to confirm compound resistance in the mutant, and rescue of the compound sensitivity in cell proliferation must be restored by expressing the fluorescently tagged protein in the mutant. Finally, it remains essential that discoveries made in *D. discoideum*, with potential for medicinal or health impacts, are validated in suitable preclinical models and then through clinical trials. These studies can include the validation of molecular mechanisms [21,192] or novel compound potency in mammalian models [21,193,194] and in clinical trials [195,196].

Despite the range of advantages in using *D. discoideum* as a research model in pharmacological research, there remain some limitations in employing the model. These limitations include the absence of many proteins associated with disease signaling pathways or as drug targets, where only 11.5% of proteins (from a subset of 287 disease-associated human proteins) show strong amino acid sequence similarity (E ≤ 10^−30^) extending over 70% of the two proteins of similar amino acid size [12]. However, this similarity is higher than *S. cerevisiae* and *S. pombe*, but lower than *D. melanogaster* or *C. elegans*. In addition, orthologous *D. discoideum* proteins have more divergent amino acid sequences and thus may not provide the same drug-binding properties. Some methodological approaches that are well-developed in mammalian models (electrophysiology) have remained difficult to develop in *D. discoideum*, and not all reagents for mammalian research (such as antibodies) are functional in the model.

## 8. Concluding Remarks

The exact pathological mechanisms underpinning many neurological disorders are yet to be determined and thus targeted therapeutics are absent. This in part is due to the lack of understanding of how certain genetic mutations or the accumulation of toxic proteins gives rise to the disorders. *D. discoideum* encodes a number of proteins homologous to proteins associated with neurological disease in humans, including many which are absent in other simple eukaryotes. *D. discoideum* can also be manipulated to express human proteins of interest, such as Tau and α-synuclein in the absence of endogenous proteins, adding to the value of the model. The ease of genetic manipulation and unique life cycle in *D. discoideum* has provided much insight into the normal and pathological roles of individual proteins implicated in these disorders. Furthermore, many of the key signalling pathways implicated in neurological disorders, such as mitochondrial and endosomal signalling, have been well characterised in *D. discoideum*. *D. discoideum* has the demonstrated potential to be employed as an early preclinical screening model to rapidly identify potentially beneficial pharmaceuticals and to elucidate the mechanisms of action of current drugs used to treat neurological disorders such as epilepsy. Despite *D. discoideum* lacking the intricate neural circuitry humans possess, it has demonstrated its value and should not be overlooked as a useful biomedical model for neurological diseases.

## Figures and Tables

**Table 1:** PD-associated genes studied in *D. discoideum*.

Human Gene	*D. discoideum* Homologue	Mutant Strain	Multicellular Phenotype	Mitochondrial Respiration	Main Findings	Ref.
*LRRK2*	*Roco* genes (*gbpC, qkgA, pats1, roco4-11*)	*gbpC-*	Decreased mound size, aberrant fruiting body morphology and decreased chemotaxis	Not tested	The Roco kinases have diverse roles in *D. discoideum* and it is yet to be determined which of the Roco proteins are true functional homologues of *LRRK2*. GbpC and Pats1 play a role in chemotaxis. Pats1 null mutants show defects in cytokinesis.	[38,39,40,41,42]
		*qkgA-*	Decreased chemotaxis	Not tested	QkgA plays a role in cell proliferation where null mutants grow quicker in shaking culture and overexpression of QkgA results in slower growth.	[38,39,40,41]
		*roco4-*	Aberrant fruiting body morphology and slug migratory defect	Elevated parameters	Deletion of Roco4 results in aberrant multicellular development and mutants display elevated mitochondrial respiratory parameters.	[38,42,43]
		*roco11-*	Aberrant fruiting body morphology	Not tested	Roco11 results in aberrant multicellular development	[38,43]
*HTRA2* *htrA*		*htra2* knockdown	Aberrant fruiting body morphology	No defect	Hyperactivity of HTRA protease activity in *D. discoideum* is lethal. HTRA localises to the mitochondria. HTRA knock-down and protease-dead HTRA strains display phenotypic defects reminiscent of mitochondrial dysfunction including altered fruiting body morphology and decreased growth rates with no endocytic defect and implicates the protease domain in these functions. Seahorse respiratory measurements indicate no defect in mitochondrial respiration in either the knock down or protease dead strains.	[44]
		*htra2* protease dead	Aberrant fruiting body morphology	No defect	HTRA knock-down and protease-dead HTRA strains display phenotypic defects reminiscent of mitochondrial dysfunction including altered fruiting body morphology and decreased growth rates with no endocytic defect and implicates the protease domain in these functions. Seahorse respiratory measurements indicate no defect in mitochondrial respiration in either the knock down or protease dead strains	[44]
*DJ-1 (Park7)* *deeJ*		*deeJ* knockdown	Aberrant fruiting body morphology	Elevated parameters	DJ-1 antisense inhibition elevates mitochondrial respiration in *D. discoideum.*	[45]
		*deeJ* knockdown exposed to H_2_O_2_	Aberrant fruiting body morphology and phototaxis (rescued by antisense AMPK)	Elevated parameters (but less elevated compared to *deeJ* knockdown alone)	Knockdown of AMPK rescues phenotypic defects observed in DJ-1 knock-down strains, suggesting that DJ-1 may play a role downstream of mitochondria by mitigating the consequences of AMPK activation.	[46]
		*deeJ* Overex-pression	No defects observed	Decreased parameters	DJ-1 overexpression inhibits mitochondrial respiration in *D. discoideum.* DJ-1 localises to the cytoplasm, and has roles in multicellular development, growth and endocytosis.	[45]
*SNCA*	No homologue. Expression of human α-synuclein in *D. discoideum*	Full length (WT) human *SNCA* C-terminally truncated human *SNCA* A53T human *SNCA*	Mild phototactic defect (not significant) Impaired phototaxis and aberrant fruiting body morphology Impaired phototaxis and thermotaxis	Elevated parameters Elevated parameters No defect	In *D. discoideum* α-synuclein was observed at the cortex and localisation was attributed to the 20 most C-terminal residues. Expression of α-synuclein caused cytotoxic phagocytosis defects. Mitochondrial respirometry parameters were elevated in strains expressing WT and truncated α-synuclein.	[13]
*Tau*	No homologue. Expression of human Tau in *Dictyostelium*	Human *Tau* (longest isoform) Co-expression of human *Tau* and full length *SNCA*	Impaired phototaxis and thermotaxis in addition to aberrant fruiting body morphology Exacerbated defects in phototaxis and fruiting body morphology when co-expressed compared to when independently expressed	Defect in complex V Normal parameters observed when co-expressed	Tau causes AMPK-dependent phototactic defects exacerbated by the co-expression of α-synuclein. Tau expression reduced axenic growth, increased *Legionella* susceptibility and impaired ATP synthesis. Tau and α-synuclein interact directly as evident by close proximity experiments. Tau and α-synuclein interact functionally in a complex relationship and when coexpressed can rescue, exacerbate or rescue the phenotypic defects evident when expressed singly. Proteomic analysis of Tau, α-synuclein and Tau/α-synuclein co-transformants revealed a distinct set of dysregulated proteins which supported functional and localisation studies.	[14]

**Table 2:** Neuronal ceroid lipofuscinoses-associated genes studied in *D. discoideum*.

Human Gene	*D. discoideum* Homologue	Localisation	Impaired Phenotypes	Interactions with Other NCL Proteins	Main Findings
*CLN1/PPT*	*ppt1*	Extracellular space [93] Macropinosome [94]	Unknown	Unknown	*ppt1* mRNA is downregulated during phagocytosis [95].
*CLN2/TPP-1*	*tpp1A* *tpp1B* *tpp1C* *tpp1D* *tpp1E* *tpp1F*	Late endosome/lysosome [96] Predicted Golgi [97]/extracellular space [93] Unknown Macropinosome [94] Unknown Endocytic compartments, Golgi complex, ER, extracellular space [97,98]	tpp1A null:precocious development, abnormal spore formation, reduced autophagy [96]. Tpp1 antisense inhibited: reduced growth, increased endocytosis, small fruiting bodies [17] Unknown Transcriptional response to *Pseudomonas aeruginosa* [99] Unknown Unknown No obvious defects in growth or development [98]	Cln3 deficiency increases *tpp1A* expression during osmotic stress [100] Interacts with Cln5 [101] Unknown Cln3 deficiency decreases *tpp1D* expression during starvation [102] Unknown Cln3 deficiency increases *tpp1F* expression and secretion during starvation [103]	Tpp1A regulates autophagy. Tpp1A mediates its effects via mTOR pathway as tpp1A antisense inhibited strains the defective phenotypes were rescued by overexpression of Rheb and mimicked by exposure to rapamycin. A suppressor of Tpp1A *(stpA)* was identified via REMI [96]. Tpp1B and Tpp1F were discovered to interact with a Golgi membrane channel (GPHR) ubiquitous to eukaryotes. This was the first time a TPP1 homologue was found to interact with GPHR [98].
*CLN3*	*cln3*	CV, endocytic compartments, Golgi complex [92]	CLN3 loss impairs aggregation, chemotaxis, multicellular development, cell adhesion, protein secretion, osmoregulation and pinocytosis. Increases proliferation [92,100,101,102,103,104]	Cln3 deficiency increases expression of CLN5, *tpp1A* and *tpp1F* but decreases *tpp1D* expression under certain conditions [100,102,103] Loss of Cln3 also reduces *grn* expression during starvation and reduces the secretion of CprA and CprB [103]	Loss of Cln3 in *Dictyostelium* results in reduced resistance to osmotic stress and reduced spore viability [100] Loss of Cln3 accelerated each stage of the multicellular developmental and this was rescued by calcium chelation [92]
*CLN4/DNAJC5*	*DDB_* *G0290017* *ddj1*	Macropinosome [94] Phagosome [105], centrosome [106] and Macropinosome [94]	Unknown Phagocytosis	Unknown Unknown	A true functional homologue of Cln4 in *Dictyostelium* is yet to be elucidated.
*CLN5*	*cln5*	CV [107], extracellular space [107], ER [101], cortex [107], macropinosome [94]	CLN5 null has reduced chemotaxis to folic acid, cell adhesion and autophagy [107]	Colocalises with CLN3 in CV [107]. Interacts with CtsD, Tpp1B [101], and is reduced in *mfsd8* null [108]	Both *D. discoideum* Cln5 and human CLN5 function as a glycoside hydrolase [101].
*CLN7/MFSD8*	*mfsd8*	Macropinosome [94]	Protein secretion [108]	Loss of *mfsd8* results in reduced secretion of Cln5 and CtsD during starvation [108]	The roles of Mfsd8 may be conserved in *D. discoideum*. In both humans and *D. discoideum* it regulates protein secretion and interacts with other NCL proteins [108].
*CLN10/CTSD*	*ctsD*	Extracellular space [103], macropinosome [94], lysosome, phagosome [109,110,111]	ctsD null causes development delay, bacterial degradation and cell death [109,112,113]	Interacts with Cln5 and reduced in *mfsd8* null [108].	Both *Dictyostelium* and human CTSD contain secretion peptides. *Dictyostelium* CtsD is highly expressed during growth and aggregation and plays similar roles in humans [108].
*CLN11/PGRN*	*grn*	Unknown	Unknown	Reduced by the loss of *cln3* during starvation [102]	
*CLN12/Park9*	*kil2*	Phagosome [114] and macropinosome [94]	Growth, defense against bacteria, fungi and metal toxicity [114,115]	Unknown	Kil2 acts as a magnesium pump and protects the cell against cation stress [114].
*CLN13/CTSF*	*cprA* *cprB* *DDB0252831*	Extracellular space [103] Extracellular space [103] Extracellular space [93,103] and endocytic vesicles [116]	Osmoregulation [117] Phagocytosis [95] Transcriptional response to *Pseudomonas aeruginosa* [99]	*cln3* deficiency reduces secretion of CprA and CprB [103]	CprA identified as the likely homologue of human CTSF [91].
*CLN14/KCTD7*	*kctd9* *DDB0238663, DDB0346929* *DDB0347398*	Unknown Unknown Macropinosome [94] Unknown	Unknown Unknown Unknown Unknown	No known interactions in *Dictyostelium* for any of the proposed homologues	Four potential homologues [8].
